# The Seed and the Metabolism Regulation

**DOI:** 10.3390/biology11020168

**Published:** 2022-01-20

**Authors:** Hayat El-Maarouf-Bouteau

**Affiliations:** Institut de Biologie Paris Seine (IBPS), Sorbonne Université, UMR 7622, 4, Place Jussieu, 75005 Paris, France; hayat.bouteau@sorbonne-universite.fr

**Keywords:** seed, hydration force, energy, germination, dormancy, ageing

## Abstract

**Simple Summary:**

Seeds are the reproductive units of higher plants. They have a significant place in agriculture and plant diversity maintenance. Because they are dehydrated, they can remain viable in the environment for centuries. This review explores the dry seed as a metabolically inactive organism, but well organized to protect its components and enter intensive repair to restore metabolic activities upon imbibition for the completion of germination. Metabolism regulation is also critical for the most important seed traits, dormancy, and ageing recovery capacity.

**Abstract:**

The seed represents a critical stage in the life cycle of flowering plants. It corresponds to a dry structure carrying the plant embryo in dormant or quiescent state. Orthodox seeds possess a very low water content, preventing biochemical reactions, especially respiration. If the desiccation of living organisms leads to a loss of homeostasis, structure, and metabolism, the seeds go through it successfully thanks to their structure, cellular organization, and growth regulation. Seeds set up a certain number of sophisticated molecules to protect valuable macromolecules or organelles from dehydration/rehydration cycles. Moreover, dormancy takes place in a coordinated process with environmental cues in order to ensure embryo development at the most appropriate conditions for the establishment of the new plant. Moreover, repair processes are programmed to be ready to operate to maximize germination success and seed longevity. This review focuses on the physiology of the seed as related to hydration forces, respiration, and biochemical reactions in the transition from thermodynamically undefined dry state to self-sustained living system. Such processes are of importance for basic knowledge of the regulation of metabolism of living organisms, but also for the control of germination in the context of climate change due to global warming.

## 1. Introduction

Seeds are important as propagation units for crops, but also for species maintenance in the natural environment. Seed germination represents the first step in the establishment of the new plant for agriculture or in natural areas. It is, therefore, important to unravel the physiological aspects of germination for basic knowledge, as well as for the good management in the context of environmental fluctuations due to global warming. Understanding germination depends on understanding the seed organization and functioning in anhydrobiosis [1]. In fact, the most important characteristic of the seeds referred to as orthodox, which are the focus of the present review, is the ability to be desiccated and to survive dry state, allowing them to be stored and distributed widely. On the contrary, recalcitrant seeds cannot tolerate dehydration. They possess a high water content and active metabolism and cannot be stored for long periods [2]. Another category with intermediate features also exists, e.g., coffee seeds, which can tolerate drying but display sensitivity to cool temperatures [3].

In the absence of metabolic activity, orthodox seeds do not meet the different definitions of living organisms. The biological definition of living organisms admitted so far is based on the ability of regeneration and the existence of metabolism. NASA’s defition of life based on thermodynamic law is a “self-sustaining chemical system capable of Darwinian evolution” [4]. In both cases, living organisms can be characterized by metabolic activity having an interaction with ecological conditions. The self-sustaining chemical system corresponds to a thermodynamic aspect of life as a system far from equilibrium [5]. Yet, the seed carries the embryo ready to live as soon as the seed rehydrates. In this review, seed organization as related to water status and seed metabolism in dry state and upon imbibition are examined to understand dormancy, germination, and ageing tolerance processes.

## 2. Dry Seed: Well-Organized to Resist

### 2.1. The Seed, a Special New Individual

The seed is composed of an embryo surrounded by reserve material and covering layers. It represents the plant dispersion organ formed by sexual reproduction as well as the new individual. The seed therefore occupies a critical position in the life cycle of the higher plant. The success of the establishment of the new individual is determined by physiological and biochemical features of the seeds in response to their environment.

**Figure 1:**
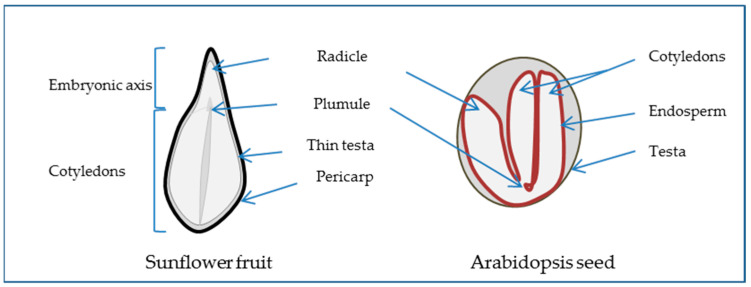
Seed morphology scheme presenting longitudinal section of sunflower fruit as a non-endospermic seed surrounded by the pericarp and *Arabidopsis thaliana* as an endospermic seed.

The angiosperm seed generally consists of the embryo, the result of the fertilization of the egg cell and one of the male pollen nuclei, the endosperm, which is the result of the fusion of the two polar nuclei with the second pollen nuclei, and the perisperm, corresponding to the nucellus and the testa or seed coat formed from the integument around the ovule. The extent to which the endosperm or perisperm persists varies between species. For example, the Arabidopsis embryo is surrounded by an endosperm layer while the sunflower embryo is not (Figure 1). When the testa is underdeveloped, the outer structure being the pericarp or fruit coat, the dispersal unit is not a seed but a fruit, as in the case of sunflower and wheat. The embryo, which represents the new individual, is comprised of the embryonic axis and one or two cotyledons. The axis includes the embryonic root (radicle), the hypocotyl, and the shoot apex (plumule). Thus, as the seed corresponds to a diverse composition of such complex tissues that have distinct developmental programs [6], studying seeds implies the study of these programs and their coordination in time and space to achieve germination [7].

Desiccation represents the last phase of seed development and corresponds to a huge loss of water content that decreases the seed water percentage in orthodox seeds to less than 10% of the dry weight (DW), depending on species (e.g., mature sunflower seeds contain 4% g H_2_O/g DW [1]). Such a low water content changes the cytoplasm from a fluid to glassy state, which severely reduces molecular diffusion and mobility, preventing chemical reactions [8]. In fact, at dry state, cellular metabolism and respiration are greatly reduced [9,10]. Thus, dry seeds maintain low levels of metabolic activity, which preserves their viability for years or even centuries, as for *Phoenix dactylifera* L. seeds [11]. The mechanisms by which the seed tolerates desiccation are discussed in specialized reviews [12,13]. In this review, the focus will be on physiological changes allowing mature seeds to successfully undertake conservation and germination.

**Figure 2:**
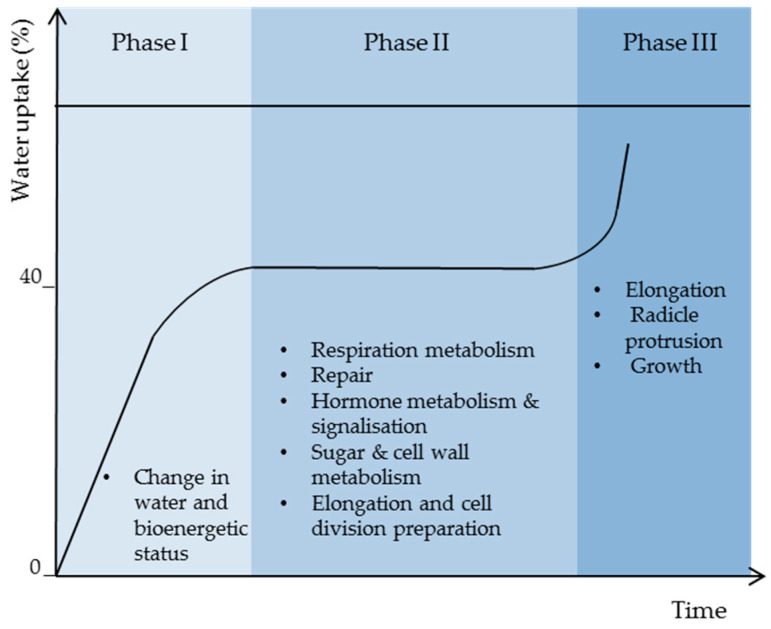
Seed imbibition curve showing the three characteristic phases with their main biological processes.

Seed germination starts with water uptake and ends with radicle protrusion. The seed water absorption rate corresponds to three phases during which controlled physiological processes take place. As shown in the Figure 2, phase I corresponds to a rapid water uptake, which induces the transformation of cell membranes from gel phase to liquid crystal state and the reorganization of cell structure and molecules required for the establishment of cell metabolism that takes place actively at constant water content corresponding to phase II (plateau phase), during which the water uptake is stopped. In fact, based on reports on different species, such as wheat, rice, Arabidopsis, and sunflower, phase II corresponds to high metabolic activity, with gene expression corresponding to respiration, hormones, sugar, and cell wall metabolism, and protein turn-over allowing repair and component preparation for cell elongation and growth [14,15,16,17,18]. During phase III, fast water uptake takes place again to ensure reserve mobilization and metabolism for root elongation and growth [19].

However, germination is not generally possible for mature seeds because they are generally dormant. They need to undergo a post-maturation phase, called after-ripening, a period that allows them to acquire the capacity to germinate. The transition of dry seeds from dormant (D) to non-dormant (ND) state corresponds to determinant physiological changes from arrested to permissive processes leading to germination. The characterization of possible chemical reactions and subsequent physiological activity at dry state remain the most difficult question in seed biology because experimental procedures require short- or long-term hydration. Yet, this question is crucial in the understanding of dormancy alleviation, germination, and longevity. 

### 2.2. Water, “Matrix of Life”

If water is the matrix of life [20], dry seeds can hardly be considered as alive and yet they bear life in the form of the embryo. Water is an essential participant in the chemistry of life by sustaining the biochemistry of the cell. It acts as a liquid and solvent for biochemical reactions, but also influences macromolecule structures [21]. Water participates in the catalytic function of proteins and nucleic acids and physically in hydrophobic associated protein folding and complex formation through the hydrogen bond [22]. Physical methods, such as thermodynamic studies or nuclear magnetic resonance spectroscopy, came to the rescue of biology for the investigation of water status in low hydrated seeds and subsequent interactions. Using thermodynamic measurement, three levels of water affinity have been characterized in pea and soybean seeds [9,23,24]. Strongly bound water was recorded at 8% of water content, weakly bound water between 8% and 24%, and very loosely bound water at contents above 24% [9]. In these ranges of water moisture, the investigation of lysozyme hydration by IR spectroscopy and heat capacity showed that with up to 0.07 g of water/g of protein, the hydration process is dominated by the interaction with charged groups. At 0.07 g/g, there is a transition in the IR spectrum and the heat capacity, reflecting a change in surface water arrangements. Between 0.07 and 0.25 g/g, most of the surface is covered with water molecules. Between 0.25 and 0.38 g/g, water condenses over the non-polar atoms not adjacent to charged or polar atoms [25]. The final stage of protein hydration is that of hydrophobic groups, which represent a large portion of the surface of the protein molecule. Water–water bonds can be then created and participate in protein–protein or protein–substrate interactions. The enzymatic activity of lysozymes becomes detectable at 0.2 g/g and changes with hydration above 0.38 g/g. Changes in the arrangement of water in the protein environment affect protein stability and enzyme properties [25,26]. On the other hand, nucleic acids require more water than proteins [25]. In fact, the end point of the hydration process of nucleic acids is about twice the level for proteins [27]. DNA structure and related biological functions are controlled by the complex dynamics of hydrating water and ions in and around the DNA [28]. It was shown that in desiccated Arabidopsis seeds the chromatin is highly condensed and can be de-condensated after hydration [29]. The property of the seed to undergo a reversible chromatin condensation/de-condensation enables to withstand desiccation and the entry in active metabolism during imbibition.

**Figure 3:**
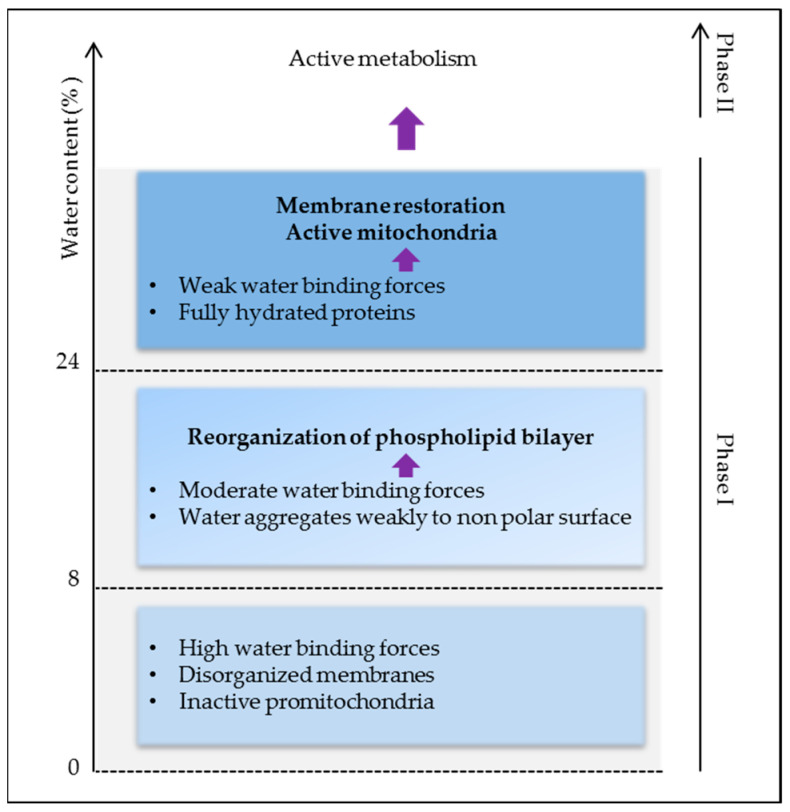
Early cellular events during imbibition as related to water binding forces.

Thus, high water binding forces in dry seeds are responsible for the lack of stability and activity of biomolecules causing low metabolism and energy. As membrane reorganization is one of the first events in the initiation of cell energization, more water (>24%) is needed to activate protein reorganization and activity for full plasma membrane and mitochondrial energy restoration (Figure 3). Indeed, below 24% of water, seed O_2_ consumption is very low and it is undetectable at around 8% [9]. Respiration plays a crucial role in providing cellular energy via oxidative phosphorylation, but it also represents the major source of reactive oxygen species (ROS) responsible for cell damage. By preventing cell metabolism, drying keeps the embryo alive, which highlights the dual role of water in life and its consumption.

### 2.3. Respiration Resumption

Holding respiration may represent the major process allowing seed longevity. This is achieved with the support of several seed features. The seed structure itself may contribute to holding respiration by O_2_ uptake limitation due to the space occupation by reserve molecules and the presence of the seed coat (for review [13,30]). More importantly, mitochondria in dry seeds are called promitochondria as their internal membranes are underdeveloped with a low number of cristae and low protein content [31,32]. Several studies using different biochemical approaches, such as adenylate pool or adenylate energy charge (AEC) ratio ((ATP + 0.5 ADP)/(AMP + ADP + ATP)), oxygen uptake, tricarboxylic cycle enzyme activity measurements, or cytological investigations, converge to state that respiration is reduced to a very low level in dry seeds and that the hydration induces an increase in mitochondria components and activity [33,34,35,36,37,38,39]. The proliferation and differentiation of mitochondria, called ‘mitochondrial biogenesis’, occur progressively upon imbibition [10,37,38]. These are considered as the prerequisite for the full reactivation of mitochondria and subsequent energy supply for germination [31,32]. However, isolated promitochondria were shown to be able to generate ATP and a membrane potential by oxidizing supplied succinate and/or NADH [31,40]. Such metabolic activity may be decisive at the onset of imbibition to help the biogenesis process to take place. Moreover, promitochondria seem to have an import apparatus ready for mitochondrial biogenesis [32]. In fact, the electron transport system is activated immediately after the initiation of imbibition and is dependent on AMP, ADP, cytochrome C oxidase, and ATPase that were recovered from dry seeds [36,41]. A recent study enabled the visualization of mitochondrial reactivation and the chondriome (all mitochondria in a cell) during imbibition [10]. They confirmed that promitochondria have reduced metabolic activity but can generate a membrane potential within the first minutes of imbibition. Further imbibition in permissive conditions for germination allowed a significant increase of mitochondrial dynamics, leading to inter-mitochondria interactions and localization around the nucleus, which may facilitate mitochondrial biogenesis and synchronization [10]. 

Mitochondrial functioning is also dependent on post-translational modifications of proteins of metabolic functions. In fact, the NADPH produced from the metabolism enables the reduction of thiol redox reactions [42]. Thus, mitochondrial resumption enables not only the production of energy as ATP for elongation and growth, but also reductants that determine redox regulation for subsequent transcription and hormonal regulation. 

### 2.4. Plasma Membrane Potential

One of the fundamental properties of living cells is the establishment of an electrical potential difference across the plasma membrane. In dry seeds, the transport of ions is not possible due to the absence of water as a conductive fluid, but also due to the loss of the integrity of membranes and their protein components. Membrane deterioration has been highlighted by the high electrolyte leakage rate measured in dry seeds and many studies have reported that seed hydration induced a membrane leakage decrease, confirming that the cell membrane was repaired upon imbibition. It was shown that the leakage decrease depends on the moisture content of the seeds, being undetectable above 24% [9]. In fact, 20% water corresponds to the minimum amount of water needed to create a hydrophilic layer that stabilizes the organization of lipids in a bilayer [43]. Such a water content corresponds to phase I of germination (Figure 3), while lipid metabolism and repair were shown to take place during phase II [44,45], suggesting the need for an intact membrane before any repair event can be started. It also corresponds to the start point of the full hydration of proteins (discussed above) that should include membrane ion channels and transporters.

At a water content less than 20%, the cell membrane may consist of fragments of a hexagonal array of hydrophilic circles formed by polar heads of phospholipids [43]. Such organization is responsible for electrolyte leakage and probably facilitates the massive entry of water at the beginning of imbibition. The characterization of these electrolytes in several seeds showed a great diversity of molecules, such as ions, amino-acid, sugars, organic acids, phenols, and phosphates, as well as hormones like gibberellic acid [43]. If the membrane disorganization of the dry seed inevitably induces the release of electrolytes at the beginning of imbibition, it would correspond to a powerful process which allows the seed to germinate on the poorest supports by modifying the external environment charges to create a membrane electrical potential.

In plants, plasma membrane potential is driven by two major components, K^+^ gradient and H^+^ ATPase activity. The plasma membrane (PM) H^+^ ATPase is responsible for membrane energization by extruding H^+^ protons, which is necessary for the activity of nutrient transporters associated to electrochemical H^+^ gradient [46]. It was demonstrated that PM H^+^ ATPase is essential for growth since the knockout of the two major PM H^+^-ATPase genes, *AHA1* and *AHA2*, is lethal in Arabidopsis embryos [47]. The role of PM H^+^ ATPase in physiological processes is regulated by post-translational modifications which correspond to the phosphorylation of C terminus residues [48,49]. It was shown that PM H^+^-ATPase presents two activity states, auto-inhibited and upregulated, depending on the coupling ratio between ATP hydrolysis and H^+^ pumping [48,50,51]. The basal state has a low coupling ratio, while the activated state has a high ratio [51]. Several signals, such as sugar or light, activate the phosphorylation of C terminus, allowing the activation state corresponding to high affinity for ATP [48]. Although H^+^-ATPase has not been actively studied in seeds, recent work has shown that high H^+^-ATPase activity was associated with germination capacity while dormant state was associated with low activity in sunflower seeds [52]. Considering that the imbibition of dry seeds is driven by the physical properties of water in the reorganization and remodeling of PM, including the proper folding of H^+^-ATPase as a protein component, and given the central role of mitochondria and reserve mobilization, the ATP/ADP ratio of the cell may be the major parameter affecting PM H^+^-ATPase activity in the seed germination process. Further investigations are needed to discover the pathways by which this protein is phosphorylated and dephosphorylated in the regulation of dormancy and germination. 

## 3. Seed Dormancy: Higher Level of Resistance 

Seed dormancy, which is the incapacity of mature seeds to germinate, is one of the most important processes in the successful establishment of the new seedling. Dormancy is finely regulated with the aim to insure germination at the optimal moment. Indeed, deep dormancy prevents field emergence and low dormancy causes sprouting. Dormancy takes place at the end of seed formation, and it’s removed during a period of several weeks to decades, called after-ripening.

### 3.1. Seed Metabolism and Dormancy

After-ripening has fascinated researchers because dormancy is alleviated in dry conditions, suggesting that some processes operate in the dry seed. Biological reactions have been investigated and transcriptional programs have been proposed to be involved in the regulation of after-ripening-mediated seed dormancy alleviation in several seeds [53,54,55,56,57]. Given the restricted molecular mobility due to the glassy state in dry seed cells, the existence of a hydrated pocket within the cell enabling gene transcription has been hypothesized [53]. To address this issue, Meimoun et al. [1] investigated transcriptomic changes after the after-ripening period in sunflower seeds using two protocols, one allowing dormancy alleviation but not the other, in order to differentiate between changes in gene expression associated with dormancy alleviation and those associated with storage only. They showed that there is no significant variation between conditions, suggesting that gene expression did not take place during after-ripening, in agreement with the absence of metabolic activity in dry seeds [1]. Furthermore, ancient studies (over 50 years) have already shown that transcription was not required for *de novo* protein synthesis in imbibed seeds, suggesting that seeds contain stored transcripts ready for use upon imbibition [58]. Since then, a number of studies have demonstrated that germination (reaching radicle protrusion) is completed in the presence of a transcription inhibitor while it is completely blocked in the presence of a translation inhibitor (for review, see [59]). This means that stored mRNAs, also called ‘long-lived mRNAs’, are necessary and sufficient to carry out the germination in sensu stricto which corresponds to the determining phase of dormancy maintenance or alleviation. 

Non-enzymatic oxidations are possible in low hydrated seeds and represent the most plausible lead to explain the observed molecular changes reported during after-ripening [60]. Indeed, mRNA oxidation was shown to be associated with dormancy release during after-ripening in sunflower and wheat [61,62], which alters the stability of stored mRNAs, being finally degraded or translated into non-functional proteins [63]. However, if a fraction of stored mRNA is inactivated, the one involved in germination has to be protected from oxidation. A recent study showed that the association of mRNA with monosomes may be the key process for mRNA preservation [64]. The identification of translated proteins from stored mRNA in rice seeds showed that they correspond to glycolysis and translation machinery, and newly synthetized mRNA are involved in pyruvate metabolism, tricarboxylic acid (TCA) cycle, or momilactone biosynthesis [65]. This indicates that these newly synthetized energy components may represent good candidates for the regulation of germination. In fact, it was shown that TCA enzyme regulation participates in the control of seed dormancy in sunflower [16]. It was also shown that TCA enzymes were thiol redox regulated and responsible for efficient TCA functioning [41]. Several other post-translational modifications, such as phosphorylation, ubiquitination, carbonylation, glycosylation, acetylation, succinylation, or sumoylation, have been proposed to play important roles in seed germination by controlling hormonal signaling, metabolism, and redox status (for review, see [66]). Carbonylation represents the most plausible modification that takes place at dry state as a consequence of the non-enzymatic generation of reactive oxygen and nitrogen species [67]. It has been shown that protein carbonylation occurs during after-ripening and may play an important role in the transition from dormant to non-dormant state in dry seeds by facilitating reserve degradation and regulating cell signaling [68]. 

Respiration and redox regulation therefore constitute the most important regulation in the initiation of the germination process, but hormonal regulation that takes place later during imbibition is also crucial to germination achievement. 

### 3.2. Internal Determinants of Dormancy

It is well established that dormancy is regulated by the hormonal balance between the main positive regulator abscisic acid (ABA) and negative ones, such as hormones like gibberellic acid (GA), ethylene (ET), auxins, or brassinosteroids, as well as some other molecules, like ROS or nitric oxide (NO). The involvement of each of them and their interactions in the whole process of germination depend on the structure of the seed and the environment. Nevertheless, ABA represents the highly conserved component of the process across species and the unique dormancy determinant as opposed to the multiple stimulants of germination. Thus, to illustrate the regulation and function of hormones in the physiology of germination, without elaborating on all the hormones and their complex signaling, the case of the ABA is the most appropriate. High ABA is induced during the maturation phase of seed development to set up desiccation and dormancy. In mature seeds, a large proportion of the stored mRNA was shown to correspond to genes in which the promoters are targets of ABA-responsive transcription factors, which could be the residual consequence of the ABA induction in the maturation phase or a regulated process to insure the execution of ABA signaling upon imbibition [69]. Upon imbibition, ABA content declines similarly in ND and D seeds during the early phase of germination, but this decrease continues in ND seeds while subsequent *de novo* ABA synthesis occurred in imbibed D seeds, leading to dormancy maintenance [70]. Therefore, ABA biosynthesis, catalysis enzymes, and corresponding genes, nine-cis-epoxycarotenoid dioxygenase (NCED) and cytochrome P450 707A (CYP707A), respectively, represent the major determinants of seed dormancy. However, a decrease in ABA content is not a prerequisite for germination as ABA signaling events represent another level of regulation [71]. The responsiveness of seeds to ABA is called ABA sensitivity and it involves several promoters, genes, and protein regulations. In recent studies, a number of these key players have been characterized in a complex network partly connected with other hormones having a dual role in this process [72,73]. However, how such players operate to arrest expansion of the embryo and growth remains unsolved. Considering the challenging energy demand of the germination process, reserve breakdown and respiration may represent the regulatory mechanisms. ABA treatment is able to inhibit reserve mobilization and sugar treatment to overcome the exogenous ABA inhibition of germination. However, the effect of endogenous ABA is still unclear [74]. Mitochondria play a central role in energy supply and they are also associated with ABA sensitivity based on works showing that several mutants of RNA processing for subunits of the electron chain display reduced ABA sensitivity. This regulation involves retrograde, anterograde, and inter-organelle signals in the transcription control of the ABA biosynthesis gene, NCED [75]. On the other hand, Paszkiewicz et al. [10] have shown that mitochondrial dynamics associated with germination condition was slightly affected by ABA treatment, arguing that mitochondrion reactivation depends only on the physical conditions of hydration and temperature. Based on these works, the optimal differentiation and functioning of mitochondrion are associated with an ABA sensitivity decrease. Accordingly, it is easy to consider that in dormant seeds, the impairment of mitochondrial activity occurs. However, it has long been established that inhibitors of oxidative phosphorylation such as cyanid can break dormancy. This paradox has still not been elucidated. The activation of the pentose phosphate pathway, the metabolic pathway that supplies reducing energy to cells, has been the most plausible hypothesis proposed [76]. Indeed, in reduced mitochondrial activity, glycolysis is activated to obtain ATP, a phenomenon known the “Pasteur effect”, leading to pyruvate production and the accumulation of fermentation by-products. Thus, anaerobic metabolism facilitates reserve breakdown and it might operate, in normal conditions, at the onset of germination when the mitochondria are not yet fully reactivated. All these data point to the importance of cell metabolism and energy regulation for successful germination.

**Figure 4:**
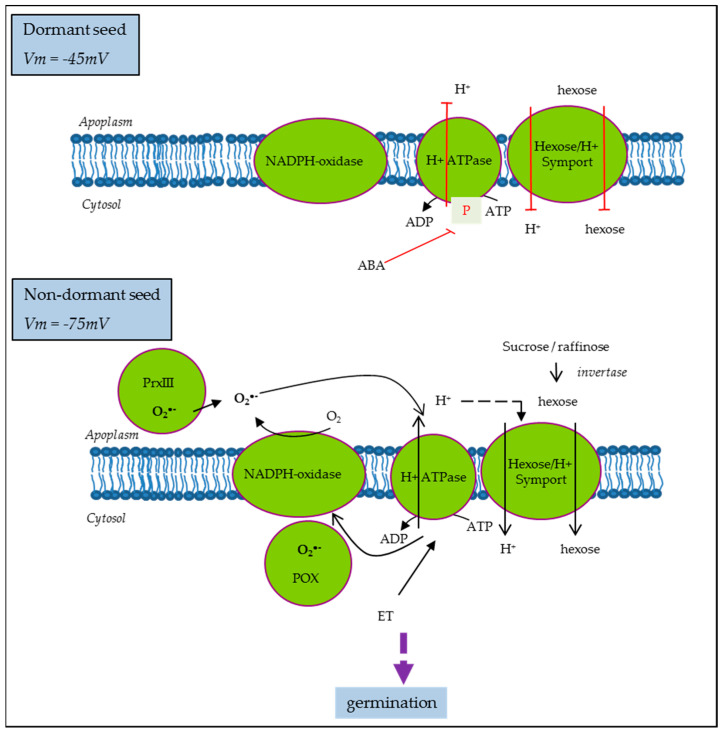
A model for seed cell polarization regulation in the control of dormancy in sunflower [52]. ABA, abscisic acid; PrxII, cell wall peroxidase III; POX, cytosolic peroxidase; ET, ethylene; Vm, PM potential.

On the other hand, it was proposed that the ABA inhibition of growth in germinating Arabidopsis seeds is driven by its inhibitory action on PM H^+^-ATPase activity [77]. ABA inhibition was less effective in Arabidopsis mutants with increased capacity for H^+^ efflux, suggesting that cytosolic acidification due to reduced H^+^-ATPase activity was the main mechanism driving growth inhibition [77]. Similarly, in sunflower, ABA induced the inhibition of PM H^+^-ATPase in non-dormant seeds, which display hyperpolarization and subsequent membrane energization. Meanwhile, in dormant seeds, PM H^+^-ATPase activity was reduced even if the corresponding proteins were present and the levels of ATP were comparable to that on ND [52]. PM H^+^-ATPase activity is also regulated by ROS and ethylene in the opposite way [52], as proposed in the model presented Figure 4. Moreover, ND cell hyperpolarization allows sugar influx through H^+^/sugar symporter [52] and it was shown that glucose and fructose contents were higher in ND as compared to D seeds in the same seed model [17]. Such sugar influx along with other solutes such as K^+^ driven by the proton motive force of PM H^+^-ATPase activity could be the prerequisite for dormancy alleviation and germination as membrane energization represents the starting point for metabolism resumption by influencing water and hexose movement, but also ions and particularly protons influencing mitochondrial activity. 

At the scale of the whole organ, specialized tissues play a critical role upon imbibition. The most striking events are the transport of hormones from/to the different tissues of the seed and consequently their specific contribution in each tissue to regulating seed germination. For example, in endospermic seeds, ABA is produced in the endosperm and transported to the embryo, while GA goes in the opposite way [78]. Moreover, cell wall loosening and programmed cell death occur specifically in the endosperm to facilitate root protrusion [79]. In non-endospermic seeds, very scarce information is available but a recent study has shown a differentiated localization of ABA, GA, and ethylene in the meristematic zone as compared to the other parts of the seed [71]. How such tissues are differentially programmed to fulfill their respective roles and what biological structure or genetic program confers them their ability, are yet to be discovered. However, whatever the state of the seed, the signal by which it awakens comes from the environment.

### 3.3. Environmental Impact on Dormancy

Environmental factors are of high importance in the awakening of the seed. Their effect on seed performance was shown to surpass genetic impact [80]. Temperature and soil moisture oscillations are the major players under natural conditions. Indeed, alternating temperatures more than constant ones can promote germination via the interplay between ROS signaling and hormones [81]. In fact, it has been shown that fluctuating temperatures alleviate dormancy by reducing ABA synthesis and signaling [82]. In the absence of a change in ABA content, there was a decrease in ABA sensitivity in sunflower dormant seeds in response to constant temperature that induced dormancy alleviation [71]. The activity of several enzymes of TCA and glycolysis were shown to be altered in the same model [16]. In wheat, high temperature treatment during seed development affects mitochondrion functioning by reducing the respiration rate and ATP content [83]. Indeed, elevated temperatures experienced by the mother plant during seed development and maturation had a negative effect on seed composition, germination, and vigor (for review, see [84]). On the other hand, light and nitrate also play important roles. Their effects are associated in dormancy cycling [85,86,87]. Seed sensitivity to both of them depends on the season and depth of dormancy. A low concentration of nitrate (around 0.1 mM) is able to promote seed germination in several species [85]. Several evidences converge towards nitrate induction of *CYP707A2* leading to ABA decrease more than GA biosynthesis in dormancy breakdown [85]. However, GA biosynthesis gene involvement has also been reported in response to environmental cues in Arabidopsis seeds from lab but also soil seed bank experiences [88,89]. Moreover, the analysis of the whole transcriptome change by nitrate treatment during seed imbibition showed the upregulation of genes involved in nitrate assimilation and transport, hormone metabolism, and energy, such as Glucose-6-phosphate dehydrogenase2, highlighting the importance of the pentose phosphate pathway [90]. Interestingly, at the level of gene expression, different environmental signals, such as light, nitrate, stratification, or after-ripening induced common changes associated to dormancy release [88]. They concern genes belonging to translation machinery, cell wall modification, and reserve mobilization. Such changes in transcript abundance are reversible, allowing the dormancy cycling phenomenon which occurs on entering a secondary dormancy when unfavorable environmental conditions are prolonged after the primary dormancy alleviation. In fact, dormancy is tightly regulated in natural conditions as in the soil, when seeds experience several scenarios of temperature, light, nitrate, and moisture, as well as microbial environment. The latter corresponds to a wide range of microbes, as pathogenic ones can induce decrease in seed longevity due to infection, and others can influence seed dormancy by breaking down the seed coat [12,91].

Thus, environmental cues influence not only seed dormancy alleviation, but determine the depth of dormancy mediated by the mother plant during seed development and maturation. The understanding of such influence is crucial for agriculture, especially in the context of environmental condition fluctuations due to global warming.

## 4. Seeds: The Ability to Recover from Ageing

Seeds remain viable, i.e., capable to germinate producing a viable plantlet, generally for a long period, from weeks to thousands of years, depending on species. Seed longevity is important for economic aspects of trade and agronomy associated with storage, but also for maintaining biodiversity. Obviously, reduced water content and metabolic activity enable such great longevity. However, long-term conservation results in a loss of viability due to deterioration processes. More specifically, unsuitable conditions of conservation, such as temperature and moisture, experienced by seeds in natural conditions or during storage accelerate deterioration processes, such as the loss of membrane integrity and oxidation of macromolecules, leading to the impairment of metabolism. However, the seed has the extraordinary ability to recover using extensive repair machinery, which represents another performance to stay alive.

### 4.1. Seed Ageing

Seed life span is of importance for field crop species impacting agriculture, but also for plant species diversity maintenance by its impact on seed longevity in the soil [92]. Seed ageing was defined as the loss of seed quality and viability over time [93]. Aged seeds germinate poorly giving abnormal seedlings or ultimately are unable to germinate. Orthodox seeds are resistant to ageing for very long time because they have a very low water content, resulting in reduced cell metabolism especially respiration which is responsible for the major production of ROS [94]. Indeed, as for the dry after-ripening process described above, enzymatic reactions and respiration are restricted by the lack of free available water preventing cellular damage. It was shown that long term storage resulting in seed loss of viability is associated with the impairment of mitochondrial activity and protein synthesis machinery [95,96]. Mitochondrion membrane integrity was identified as the primary target for ageing leading to the deregulation of its oxidative properties [97]. ROS are considered as the major cause of seed deterioration due to the oxidation of its components [98]. Lipid peroxidation has been reported in several studies on different seed species, influencing lipid metabolism and membrane integrity, as for sunflower seeds [99,100]. Furthermore, DNA damage producing double- or single- strand breaks or damaged bases is responsible for genome integrity loss and subsequent low seed quality [101]. DNA laddering has been shown in sunflower and pea aged seeds when damage extent exceeded repair capacity, pointing out the key role of mitochondria dysfunction in seed ageing [102,103]. Indeed, a direct correlation between ROS production and mitochondrial impairment leading to programed cell death has been shown in *Ulmus pumila* L. [104]. At last, total RNA content and integrity was shown to decrease in aged soybean seeds, with greater resistance of the shortest transcript (<1200 bp) mainly involved in ribosomal and translational functions, as compared to the longer transcripts (>2500 bp) corresponding to proteins with ATP-binding functions, indicating that stored mRNAs may be involved in seed longevity [105,106]. 

If cellular damage is inevitably induced during ageing, the seed can resist using several features. In fact, at the cellular level, several components protect from cell damage, such as sugars, LEA, dehydrins, or heat shock proteins involved in dehydration–rehydration protection or storage proteins being preferentially oxidized protecting vital cell components from ROS damage [92]. Indeed, in the soil, seeds experience changes in temperature and water content, two major factors that influence biochemical reaction resumption, inducing ROS production and associated damages. In addition to cellular organization, the efficacy of dormancy in preventing growth resumption and the ability for damage repair are mechanisms of importance for seed longevity in natural conditions. Such repair processes are involved in the extraordinary ability of the seed to recover from ageing. They have been explored during seed priming treatment and are of interest for all living organisms.

### 4.2. Seed Priming

Seeds possess effective repair machinery to cope with ageing-associated oxidative damage. Several non-enzymatic antioxidants have been proposed to be determinant in seed longevity, e.g., glutathione or ascorbic acid [107,108]. The antioxidant enzymes are also of importance in ROS detoxification, such as catalase, superoxide dismutase, ascorbate peroxidase, or glutathione reductase [109,110]. Other enzymes acting on specific macromolecules are also activated, such as DNA or protein repair enzymes [101,111,112]. Such machinery operates when seed hydration occurred, and its efficiency depends on the plant species and the extent of ageing damage. Based on this feature, a priming technique has been developed to improve seed quality from alterations caused by several stresses. Priming treatment consists of seed pre-hydration with a controlled amount of water which does not allow radicle elongation, i.e., a water amount corresponding phase II of the germination sensu stricto, which is sufficient to trigger the reparation processes. The addition of beneficial molecules, such as antioxidants or hormones, during priming treatment can further increase seed reparation and subsequent quality. Several priming techniques have been developed depending on the plant species and subsequent use. They all lead to an improvement of seed performance under variable environmental conditions [113]. It was shown that repair mechanisms and oxidative management in primed seeds represent the main processes associated with priming induced germination improvement, but DNA replication, cell cycle advancement, the modification of the membrane structure, and restoration of mitochondrial integrity were also proposed to explain the priming effect in germination improvement [114]. In fact, the seed engages growth preparation processes during imbibition while maintaining the desiccation tolerance machinery which allowed a successful dehydration after the priming treatment. The dried primed seed is ready to grow better, even under stress conditions. This feature is interesting for agriculture, which is why the priming technique is widely used. Priming may be even more useful in the years to come due to changing climate conditions.

## 5. Conclusions

Understanding the successful entry and exit from desiccation is fundamental for the improvement of seed germination under challenging conditions anticipated due to global warming. The application in plant germplasm conservation in seed banks is of high importance in the maintenance of genetic resources for food and environment security. In this review, several layers of regulations of seed performance were shown, from the organization and physical protection of cell components to the regulation of several signaling processes in a coordinated crosstalk. However, their implementation and the coordination of these mechanisms during seed development deserve more investigations.

## Data Availability

Not associated data marked.
